# Primary prevention of posttraumatic stress disorder: drugs and implications

**DOI:** 10.1186/s40779-015-0053-2

**Published:** 2015-10-26

**Authors:** Joachim C. Burbiel

**Affiliations:** Fraunhofer Institute for Technological Trend Analysis INT, Appelsgarten 2, 53879 Euskirchen, Germany

**Keywords:** Posttraumatic stress disorder, PTSD, Prevention, Pre-treatment, Propranolol, Prazosin, Ketamine, Morphine, Anisomycin, Osanetant

## Abstract

Because posttraumatic stress disorder (PTSD) is a highly debilitating condition, prevention is an important research topic. This article reviews possible prevention approaches that involve the administration of drugs before the traumatic event takes place. The considered approaches include drugs that address the sympathetic nervous system, drugs that interfere with the hypothalamic-pituitary-adrenal (HPA) axis, narcotics and other psychoactive drugs, as well as modulators of protein synthesis. Furthermore, some thoughts on potential ethical implications of the use of drugs for the primary prevention of PTDS are presented. While there are many barriers to overcome in this field of study, this paper concludes with a call for additional research, as there are currently no approaches that are well-suited for regular use.

## Introduction

Confrontation with extremely violent or painful events can cause a mental condition known as posttraumatic stress disorder (PTSD). This condition is highly debilitating as its symptoms – exceptionally vivid intrusive recollections, a sense of reliving the trauma, amnesia for parts of the traumatic event, and hyperreactivity to trauma reminders – severely interfere with the daily lives of the persons affected, as well as with their social environment [1]. Prevention of such a condition is thus highly desirable. Current approaches to PTSD prevention can be subdivided into primary prevention (before the traumatic event, including prevention of the event itself), secondary prevention (between the traumatic event and the development of PTSD), and tertiary prevention (after first symptoms of PTSD become apparent) [2]. This paper focuses on primary prevention by applying drugs before a traumatic event takes place. Additionally, selected literature on secondary pharmacological prevention is reviewed to derive evidence for potential primary prevention applications of additional drugs (see also [3]). Other forms of primary prevention, such as anticipative psychological interventions or measures to protect susceptible persons from being involved in potentially traumatic events, are beyond the scope of this paper.

Primary pharmacological prevention of PTSD has little relevance for average citizens, as traumatic events (e.g., severe accidents, having a child with a serious illness or events of sexualized violence) are comparatively rare and unpredictable. Accordingly, the lifetime prevalence of PTSD for the general population in six countries monitored varies, from just over 2 % in Spain and Italy, to 7.8 % in the USA and 8.8 % in Northern Ireland (France and Sweden are between those extremes). PTSD prevalence seems to be significantly influenced by the impact of armed conflicts on the population (e.g., World Wars I and II or the “troubles” in Northern Ireland) [4]. However, in the case of military personnel, experiences of extreme violence may be anticipated under certain conditions, e.g., when being deployed in combat zones. Correspondingly, PTSD is quite common among military veterans. A recent report of the US Department of Veterans Affairs states that between 2002 and 2012 approximately 257,000 veterans from Iraq and Afghanistan have been diagnosed with PTSD, out of approximately 1.63 million veterans from post-9/11 wars (16 %) [5, 6]. This is well in line with a previous comparative analysis that indicated that combat-related PTSD afflicts 4–17 % of US Iraq War veterans and 3–6 % of returning UK Iraq War veterans [7]. Furthermore, there is some evidence that deployed military health care personnel have an even higher risk of developing PTSD symptoms than other military staff [8]. PTSD thus represents a huge medical, social and financial problem to nations at war.

## Pharmacological options

Both psychological and physiological research indicates that the influence of stress on memory formation is a key point in the development of PTSD [1]. The main pharmacological options for primary PTSD prevention are thus related to stress hormones and their respective receptors. Generally speaking, poor memory formation seems to be beneficial for long-term mental health after traumatic events, while interventions that enhance memory formation (like short “de-briefings” after the traumatic event) can even raise the risk of PTSD [9].

Several biochemical systems are involved in handling acute stress, including the hypothalamic-pituitary-adrenal (HPA) axis, the autonomic nerve system (especially the sympathetic nerve system) and interactions between the pre-frontal cortex (PFC), hippocampus and amygdala. To further complicate matters, these systems show complex interdependencies. Furthermore, their roles in PTSD formation change over time between the traumatic event and the development of symptoms [10]. An overview of the pharmacological options for intervention is given in this section, roughly sorted by the biochemical systems addressed.

### Drugs targeting the sympathetic nerve system

The autonomic nerve system encompasses all nervous sub-systems that work beyond direct conscious control. It is subdivided into the sympathetic nervous system (mainly associated with activity and fear), the parasympathetic nervous system (mainly associated with rest and relaxation), and the enteric nervous system (mainly controlling the gastrointestinal system). The main transmitters associated with the sympathetic nerve system are acetylcholine, nor-adrenaline (norepinephrine) and adrenaline (epinephrine). While nor-adrenaline is physiologically short-lived and acts primarily as a short-distance messenger substance between nerve cells (i.e., a neurotransmitter), adrenaline has more hormone-like attributes in that it influences processes at a farther distance from the point of release.

The release of large amounts of adrenaline from the medulla of the adrenal gland leads to a “fight-or-flight” state, characterized by highly focused attention and the provision of energy to skeletal muscles. **Beta-blockers** inhibit the binding of adrenaline and nor-adrenaline to certain types of receptors and thus suppress the physiological and psychological effects of these stress hormones. Several dozens of different beta-blockers are currently marketed as drugs for a variety of diseases, especially hypertension and coronary artery disease (CAD). A typical example is propranolol, which was developed by Imperial Chemical Industries (ICI) in the 1960s as an anti-hypertensive. Although propranolol has a certain toxic potential, small doses are usually well-tolerated. The most dangerous undesired effect of low single doses of propranolol is triggering bronchial constriction in susceptible persons, e.g., asthmatics. Furthermore, existing heart conditions may be worsened [11]. Compared with other beta-blockers, propranolol is able to easily pass the blood–brain barrier [12]. This may be desirable in the context of PTSD prevention, as it is yet unclear whether the effect of adrenaline on memory consolidation acts both through direct action in the brain and through nervous system activity induced by binding to beta-adrenergic receptors in the periphery [13], or if only activity in the brain is of relevance [14].

The evidence concerning the effectiveness of propranolol in preventing PTSD is ambiguous. Several small studies on injured patients showed little or no effect of propranolol on the development of PTSD if given in the context of intensive care (secondary prevention). These disappointing observations, which contradict studies in animal models and pre-clinical experiments with healthy volunteers, may be attributed to three reasons: a. in the situations studied, there was a time lag between the trauma and the administration of propranolol, with the shortest gap being 2 h but more typically application of the drug began 4 to 20 h after the event (at which time crucial memory processes might already have taken place); b. the traumata of the patients tested were not sufficiently severe to spark PTSD (as recruiting heavily traumatized patients in an emergency department is nearly impossible); and c. the doses applied were too low to have an influence on memory processing [15]. However, in the case of primary prevention, the time lag issue is of no relevance, as the drug would already be present at the time of the event.

Another pressing question, especially from an ethical and legal point of view, is whether beta-blockers really inhibit memory formation (i.e., create a “clean slate”) or if they just help to control an emotional over-reaction to the trauma, leaving declarative memory formation intact. While there has been extensive research on the effect of propranolol on memory reconsolidation, which is a key factor in PTSD therapy, studies on how memory formation is influenced by propranolol in humans are scarce and inconsistent [16]. A landmark study conducted in 1994 with a small number of healthy volunteers indicated that pre-treatment with propranolol eliminates the positive effect on memory that an emotionally arousing story (accompanied by a slide show) has over an emotionally neutral account. This effect is not attributable to sedation or impaired attention, as no effect of propranolol was evident in test persons that were confronted with the neutral story only. No change in the subjective emotional reactions to the story by propranolol was observed immediately after viewing the slides. The effects of propranolol are thus not attributable to reduced subjective emotional responsiveness [17]. These results suggest that the formation of declarative memory is only brought back to a neutral level by administering propranolol. Some authors have suggested that propranolol might even enhance factual memory of traumatic events, as the strong perception of remembering highly emotional events does not necessarily correlate with realistic, detailed and accurate memory [18]. No evidence for suppressing memory consolidation below the “neutral” level by applying beta-blockers as primary preventives has been found in the literature.

Overall, it can be concluded that beta-blockers might be an option for primary prevention of PTSD from a pharmacological point of view. However, determining who should receive drugs, which drugs to administer, and the appropriate dosage would nevertheless still require substantial research efforts before any broad application could be envisaged.

**Alpha-blockers** have a mechanism of action similar to that of beta-blockers, but alpha blockers bind to a different type of receptors. A representative of this class of drugs is prazosin, which was first marketed as an anti-hypertensive by Pfizer in 1969. Since the 1990s, low doses of prazosin have been used to treat PTSD symptoms, especially sleep disturbances (like distressed awakenings) and trauma-related nightmares [19, 20]. The therapeutic effects of prazosin are usually attributed to blocking of alarm-related cognitive mechanisms mediated by nor-adrenaline (norepinephrine) in the brain [21].

There are considerably less studies on the effects of alpha-blockers on memory formation than for beta-blockers. While a study in chickens hinted that alpha-blockers might antagonize the negative effects of stress on memory formation [22], a study on rats indicated that prazosin might be useful in the primary prevention of PTSD by reducing the formation of traumatic memory [23]. The potential of alpha-blockers for PTSD prevention in humans is practically unknown [20].

While sympatholytic drugs (alpha- and beta-blockers) do not usually impair mental functions, an inherent drawback is their down-regulating activity on the cardiovascular system. While this is the desired effect when treating hypertension, it might lead to reduced peak performance in physically demanding situations, such as in combat or in emergency operations.

### Drugs interfering with the hypothalamic-pituitary-adrenal (HPA) axis

The role of corticosteroids in the development of PTSD is less straightforward than that of adrenergic transmitters and the sympathetic system. While experiments in animal models suggest that interventions that reduce the effect of glucocorticoids (e.g., blockade of glucocorticoid receptors) facilitate stress resilience, it has also been reported that individuals with lower peritraumatic cortisol levels have an increased likelihood of developing PTSD. This may be due to the fact that glucocorticoids play a role in the termination of the sympathetic stress response. Inhibiting parts of the hypothalamic-pituitary-adrenal (HPA) axis may thus increase the negative effects of adrenaline and nor-adrenaline in the development of PTSD [10].

In 2001, Schelling et al. published a study on the effect of cortisol administration to septic shock patients in an intensive care unit. This study showed that significantly fewer patients that had received cortisol in doses close to those physiologically found in persons under extreme stress had developed PTSD (1 of 9, compared with 7 of 11 in the control group). Interestingly, no effect of the treatment on the number of categories of traumatic memories could be established. Although the study was methodologically sound, the very ill patients received co-medication, including nor-adrenaline to combat the shock, the benzodiazepine midazolam, as well as the opioid fentanyl for sedation. All of these substances potentially influenced the development of PTSD. While the applied doses of midazolam and fentanyl are not known, nor-adrenaline was given for a significantly shorter time and thus in significantly lower doses to the patients of the cortisol group [24]. The findings of this study are valuable but somewhat difficult to interpret concerning the effects of the individual drugs on PTSD formation [25].

In a more controlled study in rats, glucocorticoids were administered immediately after a traumatic event. The results showed that high doses of corticoids prevented the formation of PTSD-like behavior, while low doses increased vulnerability to the trauma cue compared to placebo. From additional memory tests, the authors concluded that high doses of corticoids disrupt memory consolidation, which is the conversion of short-term memory to long-term memory, while low doses of corticoids facilitate this process [25].

In light of these exemplary studies, the findings of McCleery et al. of 2004 are still valid today: “Stress-level cortisol treatment has the theoretical advantage of correcting a possible abnormality in more vulnerable trauma victims, while not abolishing a well-functioning physiological defence mechanism in those more resilient individuals (…) who would recover without intervention. However, given the uncertainties about the status of the HPA axis in PTSD patients and the fact that acute cortisol administration has been found to enhance emotional memory, this strategy too cannot be regarded as being without risk of harm” [1].

### Other drugs

There are some indications that **opiates such as morphine** may reduce the likelihood of PTSD development if given directly after a traumatic event, but there is still little information about this effect [10]. The most important study on opiates is a *ex post* evaluation of medical records of 696 injured US military personnel that were given morphine shortly (1 h or less for 71 % of patients) after the traumatic event. These soldiers developed PTSD symptoms significantly less often than those in the control group that had not received opiates, even when other factors that might influence the outcome were taken into account [26]. Nevertheless, the pharmacological effects of opiates (e.g., sedation and disorientation) make them ill-suited for primary prevention.

An *ex post* evaluation of military personnel that had been treated for severe burns revealed that those patients who had received the dissociative anesthetic **ketamine** during surgery had significantly lower rates of PTSD than those who had not, although their injuries had been more severe than those of the control group. In this study, no correlation between PTSD and morphine equivalent units during operations was observed [27]. Again, as in opioids, the pharmacological effects of ketamine (e.g., hallucinations and psychomotor retardation) make it unsuitable for primary prevention.

Although **antidepressants** are the most commonly used drugs to treat the symptoms of PTSD, the evidence for possible secondary or even primary preventive effects is scarce [28].

Experiments on **benzodiazepines**, a class of drugs that may cause anterograde amnesia, have shown that they have no preventive effect on PTSD. Their calming effects may even facilitate memory formation in traumatic events and thus foster the development of PTSD symptoms [10, 28]. A recent meta-analysis found that benzodiazepines influence the symptoms of PTSD in such a negative way that the authors conclude that benzodiazepines should be considered relatively contraindicated for patients with PTSD or for those who have recently experienced trauma [29]. Nevertheless, the neurotransmitter system addressed by benzodiazepines (γ-amino butyric acid; GABA) should not be completely ruled out as a potential target for PTSD prevention [3].

It has been hypothesized that the conversion of labile short-term memory into enduring long-term memory requires the *de novo* synthesis of proteins. In the context of this theory, rats were injected with **anisomycin**, a protein synthesis inhibitor, immediately before or after a traumatic event. An assessment of several parameters revealed that the treated rats showed less PTSD-related behavior than did untreated rats after the same stressful event. No other behavioral differences between treated and untreated rats were observed [30]. In this context, it is worth noting that alternative mechanisms of protein synthesis inhibitors affecting memory consolidation, e.g., by triggering neurotransmitter release, have been proposed [31]. Again, experiments with protein synthesis inhibitors such as anisomycin or cycloheximide are still far away from any safe application in humans, although some of these substances have previously been used as antibiotics.

In 2014, **osanetant** was proposed as a potential drug for the secondary prevention of PTSD. This drug, originally developed as a treatment for schizophrenia but never brought to the market, antagonizes the effect of a certain neuropeptide on the neurokinin 3 receptor (NK_3_R). It was shown that its application lowers the consolidation of fear memories in mice [32]. As osanetant is safe and well-tolerated in humans, it might also be of use for the primary prevention of PTSD.

## Thoughts on ethical implications

As primary pharmacological prevention of PTSD touches issues of traumatic events and possibly even questions of life and death, ethical issues have to be considered, both in the application of pre-medication and, maybe even more, in the study of such options. Some thoughts on this are given in this section.

Concerning the application of drugs to “blue forces”, a decision balancing risks and benefits has to be taken. In this, giving drugs for the primary prevention of PTSD is very comparable to the ethical issues associated with vaccination (against natural and man-made infective agents) and “pre-treatment” against unconventional weapons (e.g., pyridostigmine against nerve agents). These considerations will strongly depend on the beneficial and adverse effects of the drugs used, and on the conditions of their application.

A further question must be asked: Will reducing the risk of developing PTSD after a traumatic event alter the disposition of commanders to expose their personnel to dangerous situations? In theory, this could be a possible effect. Nevertheless, it seems that the risk of personnel developing PTSD has little influence on tactical decisions today: The risk of developing PTSD is relatively “far away” compared to the more manifest risks of physical harm. However, lowering the risk of PTSD through drugs might alter strategic decisions, e.g., personnel rotation. This deserves further attention.

Concerning the application of chemical substances in war situations, international treaties such as the Biological Weapons Convention need to be taken into consideration. Nevertheless, these conventions address the application of pharmacological agents to the enemy. As pre-medication is given to “blue” personnel, the rules of these conventions are not applicable. Furthermore, in the case of the Biological Weapons Convention, prophylactic and protective purposes are explicitly allowed.

Research on primary pharmacological prevention of PTSD is a field struck with massive ethical issues. Given the severity of PTSD, animal models can be acceptable under controlled conditions. Nevertheless, PTSD is a complex psychological state and the comparability of such states in animal models and in humans needs to be carefully analyzed to avoid wrong conclusions. In any case, while studies in animals can give valuable clues concerning pharmacological options, they are just a first step in the development of novel therapies that must be followed up by studies with human volunteers. Such studies feature the most challenging ethical issues in this field of research: deliberately confronting volunteers with events that could lead to the development of PTSD is not an acceptable option. However, recruiting volunteers that have recently experienced a traumatic event is also ethically challenging. Furthermore, as the recruitment takes place after the event, it is by definition too late to administer drugs for primary prevention. An alternative study design could involve the pre-medication of personnel that might be confronted with traumatic situations due to their professions. This resolves the problem of deliberately applying traumatic stimuli to healthy volunteers but brings up two new issues: a. although the likelihood of traumatic events is raised for certain professions, the time of occurrence of such events is not known. This might lead to the necessity to expose healthy volunteers to adverse drug effects for a long time, with only a limited chance of positive impacts on the persons involved; and b. some of the experimental drugs can lead to diminished physical and psychological performance. This might raise the risk of occupational accidents for the volunteers (and the persons they interact with), again without direct beneficial effects for the persons involved.

## Conclusions

There is a clear indication that pharmacological secondary prevention is most effective when started early after the traumatic event. Therefore, it can be speculated that applying the same drugs before a trauma might increase effectiveness. Nevertheless, two of the most promising substances used for secondary prevention (morphine and ketamine) have pharmacological profiles that exclude their use in primary prevention, as sedation and psychotropic effects are undesirable under dangerous circumstances. Presently, sympatholytic drugs (alpha- and beta-blockers) seem to have the highest potential to be used as primary preventive drugs for PTSD, although they also have the potential both to do medical harm in average people and to deteriorate mission critical performance. While several other pharmacological options have been proposed, evidence for even a preliminary assessment is either insufficient or inconsistent in most cases (see Table 1).

**Table 1:** Tentative assessment of potential drugs for primary PTSD prevention

Class of drugs	Drugs (examples)	Currently used in PTSD therapy	Undesirable physical effects	Undesirable mental side effects	Potential effectiveness for primary PTSD prevention
Beta-blockers	propranolol	yes	some	no	yes
Alpha-blockers	prazosin	yes	some	no	unknown
Corticosteroids	cortisol	no	no	no	contradictory evidence
Opiates	morphine	no	yes	yes	yes, but strong performance impairment
Anesthetics	ketamine	no	yes	yes	yes, but strong performance impairment
Antidepressants	several sub-classes	yes	no	some	unknown
Sedatives	benzodiazepines	yes	no	yes	probably none
Protein synthesis inhibitors	anisomycin, cycloheximide	no	some	no	yes, based on animal models
NK_3_-antagonists	osanetant	no	unknown	unknown	unknown

Undesirable effects are usually dose dependent. In this table, typical therapeutic doses are the basis for the estimation of undesirable effects in healthy adults

The assessment of the potential effectiveness for primary PTSD prevention is a tentative evaluation based on currently available data

The main hypothesis about how the substances discussed in this paper prevent PTSD is by blocking the formation of long-lasting traumatic memories of an event. Apart from the pharmacological obstacles to use such “memory blockers” in primary prevention, it is also disputable whether blocking the formation of memory during missions is desirable from a tactical point of view, as individual and organizational learning from past deployments constitutes a key element of military planning. There is thus a dire need to research alternative mechanisms of primary prevention.

Studies with volunteers in the area of primary pharmacological PTSD prevention tend to be burdened by massive ethical problems. A possible direction for further clinical research would be the *ex post* evaluation of the clinical records of persons that have experienced traumatic events while being under the influence of drugs (e.g., beta-blockers or antidepressants) for medical reasons unrelated to the trauma. Such research would have to overcome several obstacles: a. incomplete medical records (military records that tend to be rather complete would be useless in this context, as military personnel suffering from conditions that require medication with such drugs would not be sent on dangerous missions); b. a large diversity of the study group, including varying post-traumatic pharmacotherapy; and c. difficult data acquisition concerning the psychological outcome several months or even years after the traumatic event. Nevertheless, such a study could reveal new and unexpected options for pharmacological primary prevention of PTSD.

## **Abbreviations**

CAD: Coronary artery disease
GABA: γ-amino butyric acid
HPA: Hypothalamic-pituitary-adrenal
ICI: Imperial Chemical Industries
NK3R: Neurokinin 3 Receptor
PFC: Pre-frontal cortex
PTSD: Posttraumatic stress disorder
